# Identification of a Five Autophagy Subtype-Related Gene Expression Pattern for Improving the Prognosis of Lung Adenocarcinoma

**DOI:** 10.3389/fcell.2021.756911

**Published:** 2021-11-18

**Authors:** Meng-Yu Zhang, Chen Huo, Jian-Yu Liu, Zhuang-E. Shi, Wen-Di Zhang, Jia-Jia Qu, Yue-Liang Yue, Yi-Qing Qu

**Affiliations:** ^1^ Department of Pulmonary and Critical Care Medicine, Qilu Hospital, Cheeloo College of Medicine, Shandong University; Shandong Key Laboratory of Infectious Respiratory Diseases, Jinan, China; ^2^ Department of Pulmonary and Critical Care Medicine, Qilu Hospital of Shandong University; Shandong Key Laboratory of Infectious Respiratory Diseases, Jinan, China

**Keywords:** lung adenocarcinoma, ARGS, risk model, SPHK1, immune cell infiltration

## Abstract

**Background:** Autophagy plays an important role in lung adenocarcinoma (LUAD). In this study, we aimed to explore the autophagy-related gene (ARG) expression pattern and to identify promising autophagy-related biomarkers to improve the prognosis of LUAD.

**Methods:** The gene expression profiles and clinical information of LUAD patients were downloaded from the Cancer Genome Atlas (TCGA), and validation cohort information was extracted from the Gene Expression Omnibus database. The Human Autophagy Database (HADb) was used to extract ARGs. Gene expression data were analyzed using the limma package and visualized using the ggplot2 package as well as the pheatmap package in R software. Functional enrichment analysis was also performed for the differentially expressed ARGs (DEARGs). Then, consensus clustering revealed autophagy-related tumor subtypes, and differentially expressed genes (DEGs) were screened according to the subtypes. Next, the univariate Cox and multivariate Cox regression analyses were used to identify independent prognostic ARGs. After overlapping DEGs and the independent prognostic ARGs, the predictive risk model was established and validated. Correlation analyses between ARGs and clinicopathological variables were also explored. Finally, the TIMER and TISIDB databases were used to further explore the correlation analysis between immune cell infiltration levels and the risk score as well as clinicopathological variables in the predictive risk model.

**Results:** A total of 222 genes from the HADb were identified as ARGs, and 28 of the 222 genes were pooled as DEARGs. The most significant GO term was autophagy (*p* = 3.05E-07), and KEGG analysis results indicated that 28 DEARGs were significantly enriched in the ErbB signaling pathway (*p* < 0.001). Then, consensus clustering analysis divided the LUAD into two clusters, and a total of 168 DEGs were identified according to cluster subtypes. Then univariate and multivariate Cox regression analyses were used to identify 12 genes that could serve as independent prognostic indicators. After overlapping 168 DEGs and 12 genes, 10 genes (ATG4A, BAK1, CAPNS1, CCR2, CTSD, EIF2AK3, ITGB1, MBTPS2, SPHK1, ST13) were selected for the further exploration of the prognostic pattern. Survival analysis results indicated that this risk model identified the prognosis (*p* = 4.379E-10). Combined with the correlation analysis results between ARGs and clinicopathological variables, five ARGs were screened as prognostic genes. Among them, SPHK1 expression levels were positively correlated with CD4^+^ T cells and dendritic cell infiltration levels.

**Conclusions:** In this study, we constructed a predictive risk model and identified a five autophagy subtype-related gene expression pattern to improve the prognosis of LUAD. Understanding the subtypes of LUAD is helpful to accurately characterize the LUAD and develop personalized treatment.

## Background

According to the most recent global cancer statistics in 2018, lung cancer is the most commonly diagnosed cancer, whose diagnosis rate has reached 11.6% (Bray et al., 2018). Lung cancer is also the leading cause of cancer death, accounting for 18.4% of all the cancer deaths (Bray et al., 2018). Lung adenocarcinoma (LUAD) is the most common subtype and accounts for more than 40% of lung cancers, and its clinical outcome still remains grim (Nakamura and Saji, 2014; Zappa Mousa, 2016). Although great advances in surgery, radiotherapy, and systemic treatment have significantly prolonged the clinical survival time of LUAD patients (Wright et al., 2006; Hirsch et al., 2017; Gettinger et al., 2018), 5-year survival rates still vary from 4% to 17% depending on the pathological TNM stage (Hirsch et al., 2017). Early detection, diagnosis, and intervention contribute to a better clinical outcome as well as the prognosis of LUAD patients, such as early diagnosis based on low-dose computed tomography, which could finally improve lung cancer mortality by nearly 20% (Aberle et al., 2011). Hence, it is essential to identify new biomarkers for early diagnosis and intervention and eventually improve the prognosis of LUAD.

Autophagy has been illustrated to be related to various cancers. Autophagy has been proven to have opposing and context-dependent roles during the process of tumorigenesis, and interventions to both stimulate and inhibit the many processes of autophagy have been proposed as a cancer therapy (Levy et al., 2017). It is clear that autophagy is a key biological process and that it is associated with tumorigenesis (Martinet et al., 2009; Dikic et al., 2010). In the very recent years, autophagy-related genes (ARGs) have been investigated in both inflammatory diseases (including pulmonary diseases) (Wang, 2015; Racanelli et al., 2018; Kim et al., 2019; Larabi et al., 2020) and various cancers (Wang et al., 2019a; Wan et al., 2019; Zhu et al., 2020a). For lung cancer, there are some newly released studies that have identified ARG prognostic signatures in LUAD and lung squamous cell carcinoma (LUSC) (Zhu et al., 2020b; Zhang et al., 2020).

Existing studies further elucidated the crucial roles of ARGs in the biological processes. ARG expression patterns not only participate in inflammasome formation but also could serve as prognostic biomarkers in cancer. Various ARGs are involved in the maintenance of intestinal homeostasis, such as ATG16L1, IRGM, LRRK2, ATG7, p62, optineurin, and TFEB (Kim et al., 2019). A six autophagy-related gene expression signature (including EIF4EBP1, TP63, BNIP3, ATIC, ERO1A, and FADD) showed better performance for predicting the survival of LUAD and LUSC patients than other clinicopathological variables (Zhu et al., 2020b). Another study constructed a risk model based on five autophagy-related gene expression levels, which could also predict the prognosis and serve as a prognostic biomarker in LUAD patients (Zhang et al., 2020). All of the above findings confirm the role of autophagy in lung cancer and indicate that ARGs could serve as prognostic biomarkers. Autophagy plays vital roles in the innate immune system and the acquired immune system, which could influence the levels of infiltrating immune cells. Take antigen presentation as an example, autophagy could not only disrupt the process but also promote the antigen presentation. One study found that NBR1 targets and degrades the MHC I, thus disrupting its antigen presentation ability to CD8^+^ T cells, which in turn can be reversed by autophagy inhibitor, for NBR1 is an autophagy cargo receptor gene (Yamamoto et al., 2020). Another two studies suggested that the activation of autophagy promotes antigen presentation to CD8+T lymphocytes mediated by dendritic cells, which then stimulate cytotoxic responses (Li et al., 2012; Wang et al., 2019b). Besides, TIM-4 can bind to AMPK-a1, activate autophagy, and degrade TAA, thereby disrupting antigen presentation and leading to a decrease in CD8^+^ T cells (Baghdadi et al., 2013). Furthermore, previous studies have illustrated that the prognostic value of tumor-infiltrating lymphocytes (TILs) significantly differs according to histological type and other factors in non-small cell lung cancer (NSCLC) patients (Kinoshita et al., 2017). Combining the previous existing studies, we found that autophagy is closely related to TILs, so we supposed that TILs may be affected by autophagy, further influencing the prognosis of LUAD patients.

In the current study, we pooled an autophagy subtype-related gene expression pattern in The Cancer Genome Atlas (TCGA) database and validated it in the Gene Expression Omnibus (GEO) database. We identified two tumor subgroups using consensus clustering analysis based on 28 prognostic ARGs, in which we found that cluster 2 had poor prognostic value in LUAD compared to cluster 1. Then, we analyzed the differentially expressed genes (DEGs) of these two clusters and performed univariate and multivariate Cox regression analyses to obtain the prognostic ARGs and overlapped the DEGs and prognostic ARGs. Finally, 10 genes were selected, and a risk model was constructed based on the coefficient value of each independent risk gene to predict the prognosis of LUAD patients. Many previously published works have simply focused either on consensus clustering analysis or on risk models. Few studies have focused on both at the same time. Therefore, in the current study, we combined both of these methods to explore the prognosis of LUAD. Furthermore, unlike existing studies, we further validated their expression and prognostic value and finally explored their associations with TILs.

## Materials and Methods

### Data Collection and Validation of Differentially Expressed ARGs in LUAD

The gene expression profiles and clinical information of LUAD patients were downloaded from the TCGA database (https://tcga-data.nci.nih.gov/tcga/). The Human Autophagy Database (HADb; http://www.autophagy.lu) was used to extract genes involved in autophagy. In detail, TCGA contains a total of 594 patients (including 59 adjacent normal lung tissues and 535 NSCLC tissues). Gene expression data from TCGA were analyzed by the limma package in R software. The independent cohort GSE72094 was downloaded from the GEO database (http://www.ncbi.nlm.nih.gov/geo/) for data validation. The dataset contains both expression levels of related genes and clinical information such as age, sex, survival status, and survival time. All the raw data including expression data and survival data from TCGA and GSE72094 are displayed in the Supplementary Information file. The significant cutoff value was *p* < 0.05 and absolute fold change >2. In addition, all expression levels of the ARGs were visualized as volcano plots using the ggplot2 package in R software. Furthermore, all DEARGs were displayed with heat maps using the pheatmap package in R software.

### Validation of 28 Significant ARGs Using Quantitative Reverse Transcription-Polymerase Chain Reaction

Human LUAD cells (A549) and normal bronchial epithelial cells (16HBE) were purchased from Cell Bank, Institute of Life Sciences, Chinese Academy of Sciences Cell Bank (Shanghai, China) and confirmed by short tandem repeat (STR) profiling. Total RNA was extracted from 16HBE and A549 cells using TRIzol reagent (Invitrogen, Carlsbad, CA, USA). After the purity and concentration of the total RNA were determined, the total RNA was reverse transcribed into cDNA using the PrimeScript RT Reagent Kit (Accurate Biology). The qRT-PCR was performed using the SYBR Green Premix Ex Taq II (Accurate Biology). The PCR conditions were set as follows: 95°C for 30 s, followed by 40 cycles at 95°C for 5 s and 60°C for 30 s for each specific primer. Finally, the relative mRNA expression levels of 28 genes were calculated using the 2^−ΔΔCT^ method. The primer sequences are listed in a Supplementary Table S1.

### Functional Enrichment Analysis

Functional enrichment analysis includes Gene Ontology (GO) and Kyoto Encyclopedia of Genes and Genomes (KEGG) in this study. In brief, GO enrichment analyses predict the function of the target genes and KEGG is a widely used database for systematic signaling pathway analysis according to gene functions. In this study, the clusterProfiler package in R software was used to perform the functional enrichment analysis of DEARGs in LUAD, and GOplot package in R software was employed to visualize all of the enrichment analysis results. The identification criterion of significant GO terms and KEGG pathways was *p* < 0.050.

### Establishment of the Risk Model Based on Prognostic ARGs in LUAD

Consensus clustering analysis of the DEARGs inferred the optimal number of clusters, the lowest proportion of ambiguous clustering, and the best cumulative distribution function (CDF) value by taking the k value of 2. Finally, two clusters were identified and DEGs were analyzed. The Cox regression analysis, also called the proportional hazards model, chooses survival outcomes and survival time as dependent variables. This model not only analyzes the impact of many factors on survival at the same time but also analyzes data with censored survival time and does not require estimation of the survival distribution type of the data. In our present study, univariate Cox proportional hazard regression analysis was used to identify ARGs associated with overall survival (OS), which were selected as prognostic biomarkers and used for further multivariate Cox regression analysis. According to the multivariate Cox regression analysis and overlapping with DEGs based on the two clusters, 10 independent prognostic ARGs were identified. At the same time, the regression coefficient and hazard ratios (HRs) were also calculated using multivariate Cox regression analysis, and the coefficient value and the gene expression levels were used to construct the risk model based on the risk score. Finally, the median risk score was the cutoff value, dividing all of the LUAD patients into low-risk and high-risk groups. According to our description above, we used both univariate and multivariate Cox regression analyses to further investigate whether these identified ARGs could serve as independent prognostic factors. In addition, we pooled all LUAD patients with complete clinical information and calculated all expression levels and risk scores of prognostic ARGs to explore the value of the constructed risk model. Receiver operating characteristic (ROC) curve analysis was used to evaluate the predictive accuracy of our risk model.

### Correlation Analysis Between ARGs and Clinicopathological Variables in LUAD

After screening ARGs and exploring their associations with OS, we moved our attention to the relationship between prognostic ARGs and clinicopathological variables. Therefore, in this study, correlation analysis was performed to further explore the correlation between prognostic ARGs and clinicopathological variables in LUAD, including age (≤65 years group and >65 years group), sex (female and male groups), stage (I&II and III&IV groups), pathological T stage (T1&T2 and T3&T4 groups), pathological N stage (N0 and N1–N3 groups), and pathological M stage (M0 and M1 groups).

### Validation of Prognostic ARGs in LUAD

Prognostic ARGs were validated using two public databases, Gene Expression Profiling Interactive Analysis (GEPIA) (http://gepia.cancer-pku.cn) and Kaplan–Meier Plotter (https://kmplot.com/analysis/). The GEPIA database contains information on a variety of cancers, including LUAD, and has expression data, survival data, and exact clinical stage data. The Kaplan–Meier plotter is a widely accepted and widely used online tool used to explore the survival rates of one and a list of genes as well as non-coding RNAs, including the survival time, survival status, clinical stages, and smoking histories. Therefore, in this study, these two databases were used to verify the prognostic value of the five well-explored prognostic ARGs.

### Exploration of the Immune-Related Mechanism of Sphingosine Kinase 1

The immune-related mechanism of SPHK1 was explored using two databases, including TISIDB (http://cis.hku.hk/TISIDB/) and Tumor IMmune Estimation Resource (TIMER) (https://cistrome.shinyapps.io/timer/). In general, as the official website information, TISIDB is an integrated repository portal for tumor-immune system interactions. According to this database, we could explore the interaction between tumors and immunity because it is a powerful website containing a large amount of tumor immunity-related data. Meanwhile, TIMER is a comprehensive resource for systematic analysis of immune infiltrates across diverse cancer types, including LUAD. This database contains six kinds of immune cell infiltrates, including B cells, CD4^+^ T cells, CD8^+^ T cells, neutrophils, macrophages, and dendritic cells. All immune cell infiltrate levels were calculated using the TIMER algorithm. Finally, the resulting figures were dynamically displayed to conveniently assess the tumor immunological, clinical, and genomic features.

In our present study, we validated SPHK1 expression levels and its relationship with OS in both of these databases. Then, we further explored OS between low immune cell infiltration levels and high immune cell infiltration levels in TIMER. Next, correlation analysis between SPHK1 and immune cell infiltration levels was performed using both the TIMER and TISIDB databases. Furthermore, the correlation between somatic copy number variation (SCNA) levels of SPHK1 and immune cell infiltration levels was also determined. The purity-corrected partial Spearman method was used to analyze the data.

### Statistical Analysis

Statistical analysis was performed using R software, and *p* < 0.050 was regarded as statistically significant. We divided patients into high-risk and low-risk groups of the 10 ARGs based on risk scores. Single comparisons of the expression rates between the two groups were performed using Student’s t-test. The unpaired *t* test was used to assess expression levels of the ARGs between the high-expression and low-expression groups. Kaplan–Meier survival curves were generated for the TCGA cohort and GSE72094 and analyzed using the log-rank test. The correlation between gene expression levels and infiltrating immune cell levels was determined using the purity-corrected partial Spearman method.

## Results

### Identification and Functional Enrichment Analysis of Differentially Expressed ARGs in Lung Adenocarcinoma From Cancer Genome Atlas Database

**FIGURE 1:**
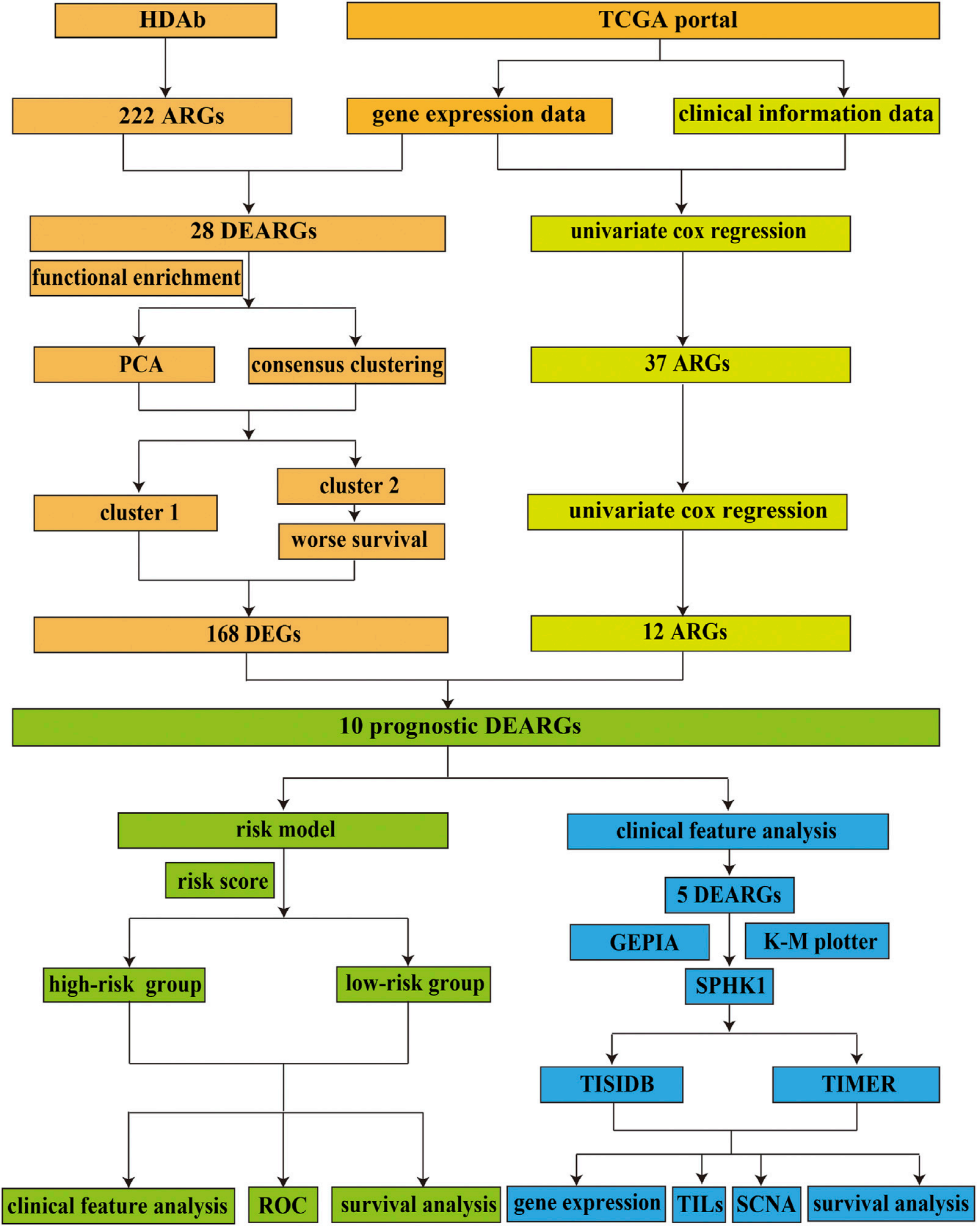
Flowchart of the current study. HADb, Human Autophagy Database; TCGA, the Cancer Genome Atlas; ARGs, autophagy-related genes; DEARGs, differentially expressed autophagy-related genes; PCA, principal component analysis; DEGs, differentially expressed genes; ROC, receiver operating characteristic; GEPIA, Gene Expression Profiling Interactive Analysis; K-M plotter, Kaplan–Meier plotter; TILs, tumor-infiltrating lymphocytes; SCNA, somatic copy number variation.

**FIGURE 2:**
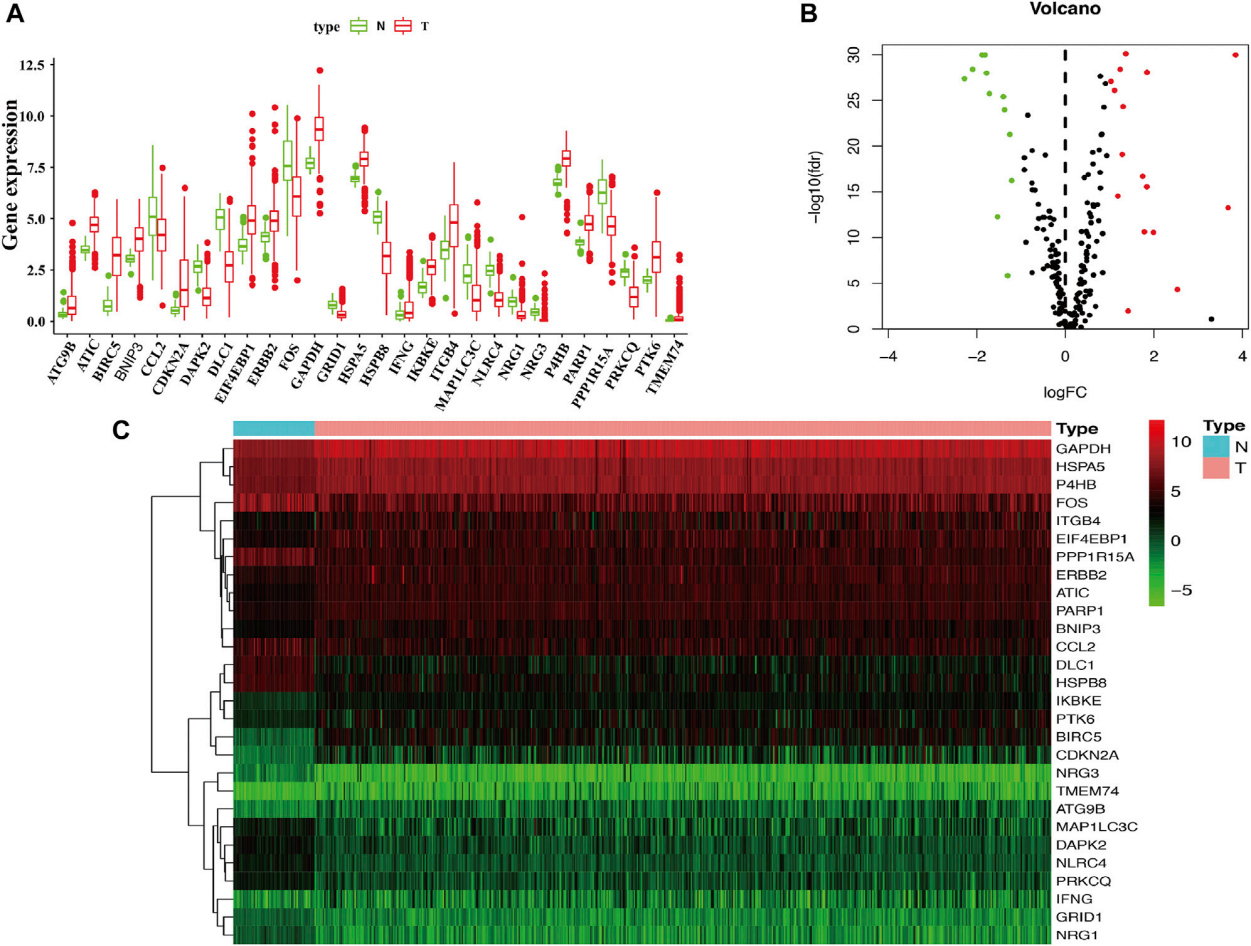
Identification of differentially expressed ARGs in LUAD. **(A)** The boxplot of the differentially expressed ARGs. The red color indicates tumor tissues, and the green color indicated the non-tumor. **(B)** Volcano plot of the 222 ARGs analysis. There were 28 ARGs differentially expressed in LUAD, including 12 downregulated genes and 16 upregulated genes. X-axis: log 2-fold change; Y-axis: −log10 fdr for each probe. **(C)** Two-dimensional hierarchical clustering of the significant 28 differentially expressed ARGs in LUAD patients. LUAD, lung adenocarcinoma.

The flow diagram of this study is shown in Figure 1. In this study, gene expression profiles from TCGA database in LUAD were selected, and a total of 222 ARGs from HADb were identified (Supplementary Table S1). Genes with *p* < 0.05 and absolute fold change >2 were considered DEARGs. Finally, a total of 28 ARGs were pooled that were differentially expressed in LUAD, including 12 downregulated genes and 16 upregulated genes (Figure 2) (Supplementary Table S1).

**FIGURE 3:**
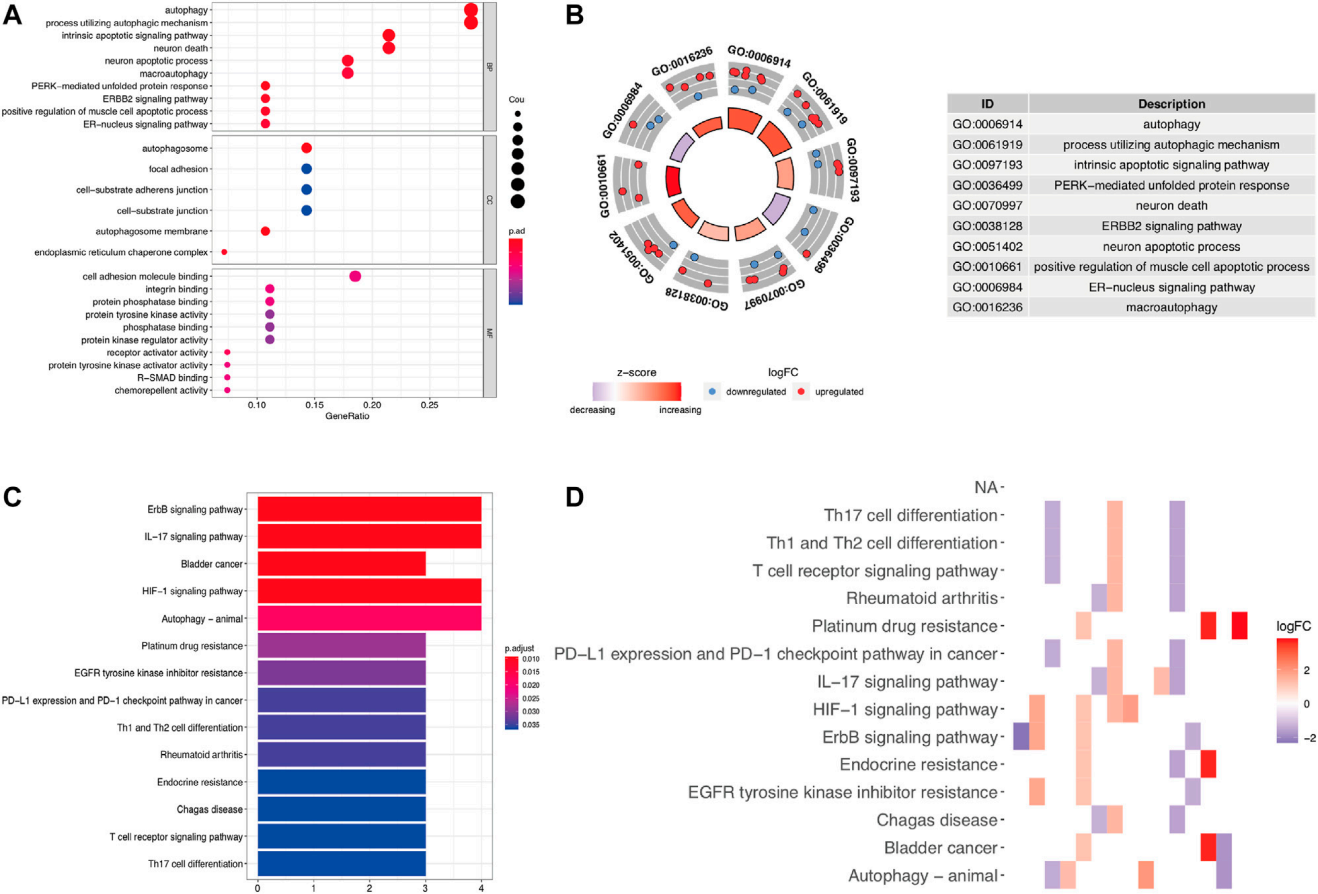
Functional enrichment analysis of differentially expressed ARGs. **(A,B)** The bubble plot and circle plot of significant GO terms. **(C,D)** The bar plot and heat map of enriched KEGG pathways. GO, Gene Ontology; KEGG, Kyoto Encyclopedia of Genes and Genomes.

To determine the biological functions of the 28 DEARGs, gene ontology and KEGG enrichment analyses were performed. The top five associated GO terms were autophagy (*p* = 3.05E-07), process utilizing autophagic mechanism (*p* = 3.05E-07), intrinsic apoptotic signaling pathway (*p* = 3.75E-06), neuron death (*p* = 9.09E-06), and neuron apoptotic process (*p* = 2.22E-05) according to both the functioned gene numbers and *p*-value (Figures 3A,B). Furthermore, all of these top five items were involved in biological processes, indicating that these 28 DEARGs participated in the biological processes of LUAD. In addition, the top 25 GO terms are shown in Table 1. Further KEGG analysis results indicated that the 28 ARGs were significantly enriched in the ErbB signaling pathway, IL-17 signaling pathway, and bladder cancer (all *p* < 0.001) (Figure 3C), and they were involved in a total of 14 KEGG pathways (Figure 3D). According to the results of functional enrichment of 28 DEARGs, we found that they were not only connected to autophagy but also involved in other biological processes. Therefore, in this study, we pooled the specific roles of ARGs in both autophagy and LUAD.

**TABLE 1:** Top 25 GO terms based on 28 differentially expressed autophagy-related genes.

Ontology	ID	Description	*p* value	Count
BP	GO:0006914	Autophagy	3.05E-07	8
BP	GO:0061919	Process utilizing autophagic mechanism	3.05E-07	8
BP	GO:0097193	Intrinsic apoptotic signaling pathway	3.75E-06	6
BP	GO:0036499	PERK-mediated unfolded protein response	4.06E-06	3
BP	GO:0070997	Neuron death	9.09E-06	6
BP	GO:0038128	ERBB2 signaling pathway	1.36E-05	3
BP	GO:0051402	Neuron apoptotic process	2.22E-05	5
BP	GO:0010661	Positive regulation of muscle cell apoptotic process	2.53E-05	3
BP	GO:0006984	ER-nucleus signaling pathway	4.52E-05	3
CC	GO:0005776	Autophagosome	7.39E-06	4
CC	GO:0000421	Autophagosome membrane	1.25E-05	3
CC	GO:0034663	Endoplasmic reticulum chaperone complex	0.000106742	2
CC	GO:0005925	Focal adhesion	0.002391067	4
CC	GO:0005924	Cell–substrate adherens junction	0.00245631	4
CC	GO:0030055	Cell–substrate junction	0.002545205	4
MF	GO:0030546	Receptor activator activity	0.000100852	2
MF	GO:0030296	Protein tyrosine kinase activator activity	0.000340315	2
MF	GO:0070412	R-SMAD binding	0.000560092	2
MF	GO:0050839	Cell adhesion molecule binding	0.000761564	5
MF	GO:0045499	Chemorepellent activity	0.000774116	2
MF	GO:0005178	Integrin binding	0.000919404	3
MF	GO:0019903	Protein phosphatase binding	0.001074992	3
MF	GO:0004713	Protein tyrosine kinase activity	0.002436602	3
MF	GO:0019902	Phosphatase binding	0.002475853	3
MF	GO:0019887	Protein kinase regulator activity	0.00255553	3

### Identification of 2 Clusters Using Consensus Clustering and the Differentially Expressed Genes Shared Between These 2 Clusters in Lung Adenocarcinoma

**FIGURE 4:**
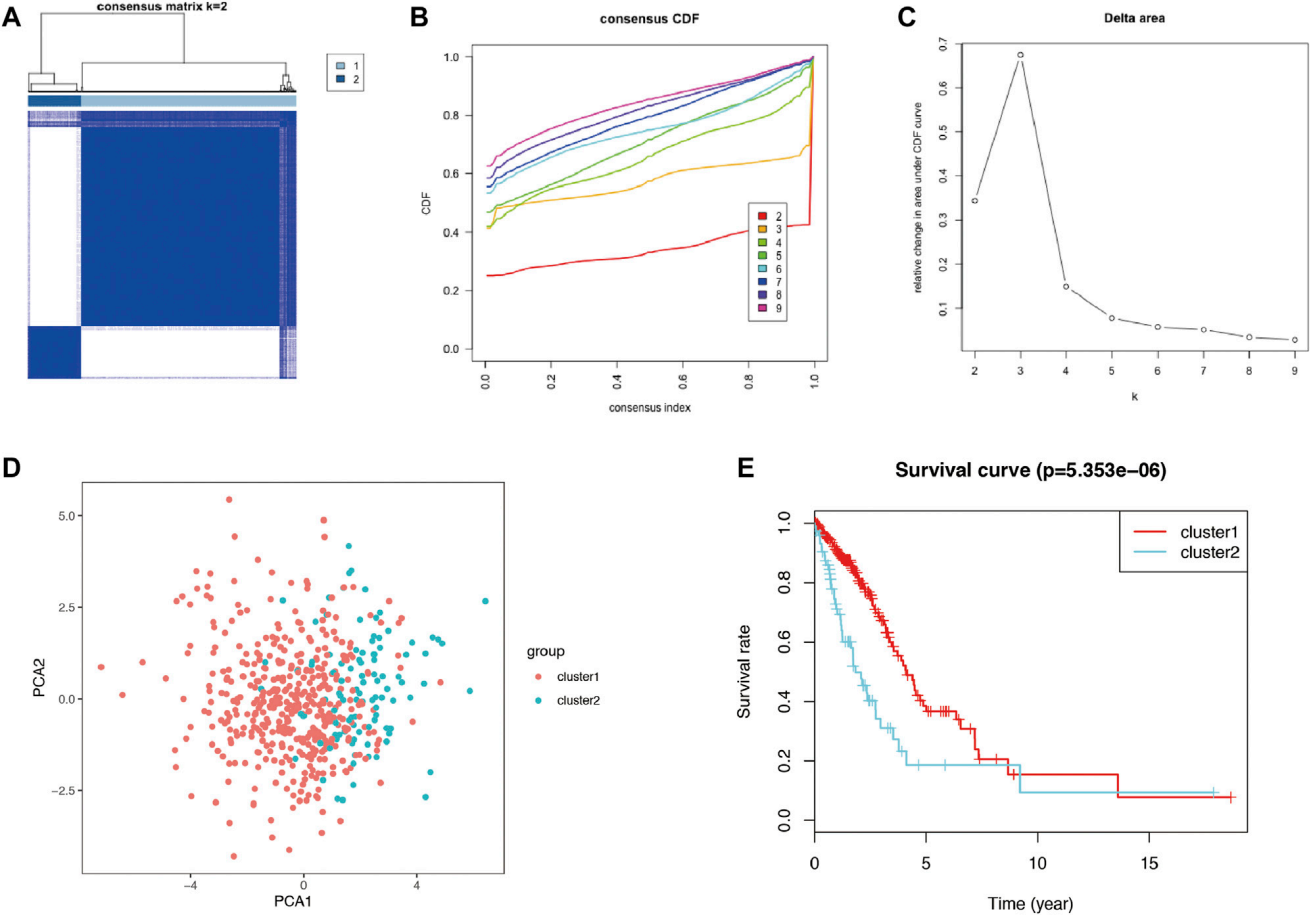
The consensus clustering analysis and the principle components analysis. **(A–C)** The consensus clustering analysis of the 28-prognostic ARGs, inferring the optimal number of clusters, the lowest proportion of ambiguous clustering, and the best CDF value by taking the k value of 2. **(D)** The principle component analysis of the prognostic autophagy-related genes in LUAD patients. **(E)** Kaplan–Meier analysis between cluster 1 and cluster 2. ARGs, autophagy-related genes; CDF, cumulative distribution function; LUAD, lung adenocarcinoma.

Autophagy may exhibit different expression patterns among LUAD patients, potentially affecting the prognosis and gene expression signature. In this study, 28 DEARGs were used to identify autophagy subtypes associated with the overall survival of LUAD. Consensus clustering was used to explore the similarity of 28 DEARGs’ expression patterns. By selecting a k value of 2, we obtained the optimal CDF value and classified the LUAD patients into two clusters (Figures 4A,C). Principal component analysis (PCA) results revealed two significantly different distribution patterns of LUAD patients. The samples of cluster 1 and cluster 2 were distributed on the left side and right sides, respectively (Figure 4D). Consensus clustering and principal component analysis suggested that autophagy may play a role in the occurrence and development of LUAD. In addition, to explore whether these two clusters of gene expression levels affect clinical outcomes, we constructed a prognostic classifier using Kaplan–Meier analysis. The results revealed that the prognosis of cluster 1 was better than that of cluster 2 (*p* < 0.001) (Figure 4E). Furthermore, since different clusters have shown variations in autophagy-related genes and patient prognosis, we explored the DEGs between cluster 1 and cluster 2. A total of 168 DEGs (76 upregulated genes in cluster 1 and 92 upregulated genes in cluster 2) were screened (Supplementary Table S1).

### Establishment of the Risk Model Based on the Autophagy-Related Genes and Differentially Expressed Genes Between Cluster 1 and Cluster 2 to Improve the Prognostic Prediction of Lung Adenocarcinoma

**FIGURE 5:**
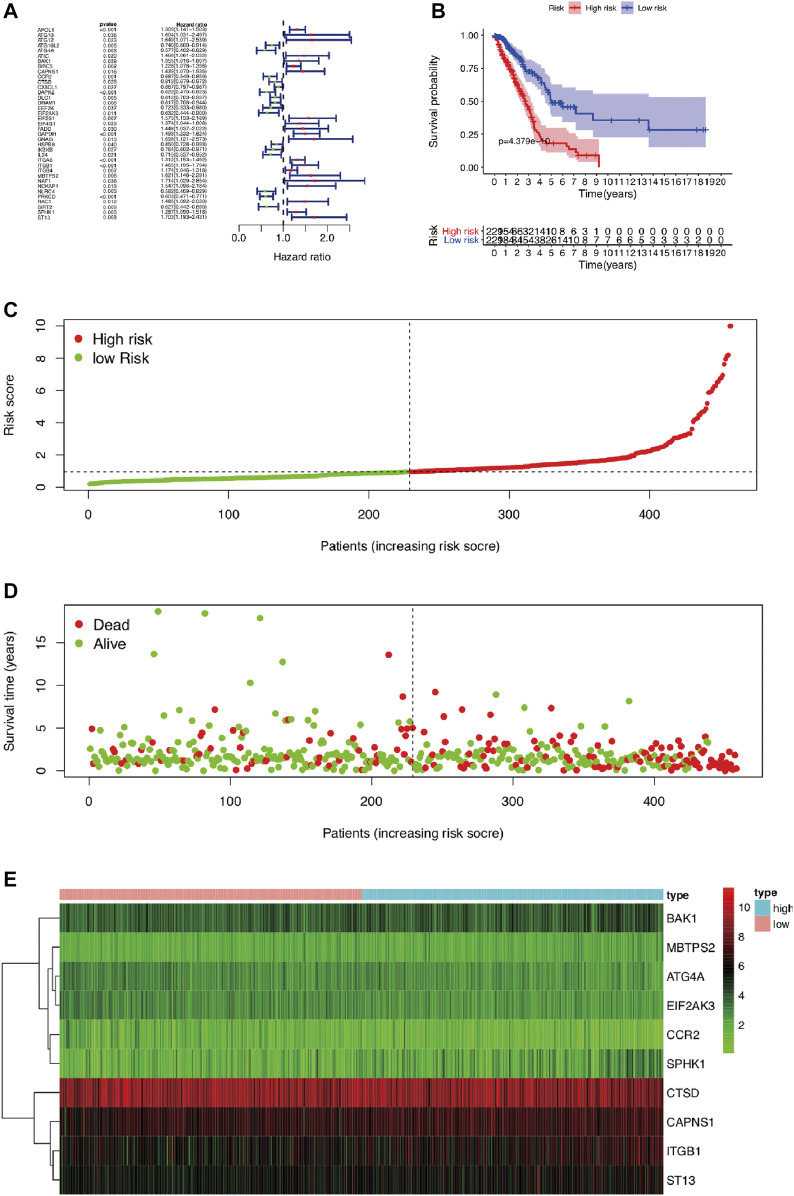
The prognostic risk model based on ARGs in LUAD. **(A)** The univariate Cox regression analysis screened prognosis-related genes. **(B)** Kaplan–Meier analysis of OS between high-risk and low-risk groups in LUAD patients based on risk scores according to 12 autophagy-related gene signatures. **(C)** Visualization of the risk score plot of high-risk and low-risk groups in LUAD patients. **(D)** Survival status was displayed in red and green dots based on the risk model as the risk score increasing in LUAD patients. **(E)** Two-dimensional hierarchical clustering of the significant 10 differentially expressed prognostic ARGs in high-risk and low-risk LUAD patients. ARGs, autophagy-related genes; LUAD, lung adenocarcinoma; OS, overall survival.

To reveal the distinct expression signature of ARGs, we constructed a risk model to predict the prognosis of LUAD. First, univariate Cox regression analysis was used to identify the prognostic ARGs, and 37 ARGs were pooled as prognostic factors, among which 16 of 37 were identified as protective factors (HR < 1), while another 21 genes were identified as risk factors (HR > 1) (Figure 5A). Then, multivariate Cox regression analysis was conducted, and the results suggested that 12 genes (APOL1, ATG12, ATG4A, BAK1, CAPNS1, CCR2, CTSD, EIF2AK3, ITGB1, MBTPS2, SPHK1, ST13) represented independent prognostic indicators, which were selected for further exploration of the prognostic pattern (Table 2). Based on the previous important clustering, we overlapped the 12 independent prognostic indicators and 168 DEGs between cluster 1 and cluster 2. Finally, 10 genes (ATG4A, BAK1, CAPNS1, CCR2, CTSD, EIF2AK3, ITGB1, MBTPS2, SPHK1, ST13) were selected for further analysis. Subsequently, the coefficient value of each independent risk gene was calculated, and our prognostic model based on the 10 genes was formed as follows: risk score = (−0.579 × ATG4A expression) + (0.224 × BAK1 expression) + (0.294 × CAPNS1 expression) + (−0.345 × CCR2 expression) + (−0.165 × CTSD expression) + (−0.561 × EIF2AK3 expression) + (0.230 × ITGB1 expression) + (0.479 × MBTPS2 expression) + (0.166 × SPHK1 expression) + (0.317 × ST13 expression). According to this formula, we calculated the risk score of each patient, and all of LUAD patients were divided into low-risk (*n* = 229) and high-risk groups (*n* = 229). Survival analysis results indicated that there was a difference between the high-risk and low-risk groups, and the low-risk group exhibited a significantly better prognosis than the high-risk group (*p* = 4.379E-10) (Figure 5B). The risk score plot, survival time, and status plot are shown in Figures 5C,D. In addition, these 10 independent risk genes are displayed in a heat map to show the different expression levels between the high-risk and low-risk groups (Figure 5E).

**TABLE 2:** Twelve autophagy-related genes are independent prognostic indicators using multivariate Cox regression analysis.

Gene	coef	HR	HR.95L	HR.95H	*p* value
APOL1	0.1239302	1.1319369	0.9835550	1.3027041	0.0838679
ATG12	0.7672543	2.1538443	1.3382951	3.4663844	0.0015767
ATG4A	−0.579587	0.5601290	0.3620170	0.8666568	0.0092520
BAK1	0.2235042	1.2504509	0.9328130	1.6762497	0.1349648
CAPNS1	0.2937850	1.3414955	0.9570607	1.8803512	0.0881531
CCR2	−0.344522	0.7085589	0.5469677	0.9178890	0.0090880
CTSD	−0.164576	0.8482525	0.6898705	1.0429961	0.1185853
EIF2AK3	−0.560724	0.5707951	0.3685623	0.8839945	0.0119893
ITGB1	0.2298494	1.2584104	0.9652966	1.6405287	0.0893375
MBTPS2	0.4789932	1.6144482	1.0610497	2.4564759	0.0253076
SPHK1	0.1659534	1.1805181	0.9692465	1.4378416	0.0990470
ST13	0.3170819	1.3731150	0.9276819	2.0324260	0.1130160

HR, hazard ratio.

### Validation of the 28 Differentially Expressed Autophagy-Related Genes Expression Pattern and Prognostic Value of the Risk Model Using Quantitative Reverse Transcription-Polymerase Chain Reaction and an Independent Cohort

**FIGURE 6:**
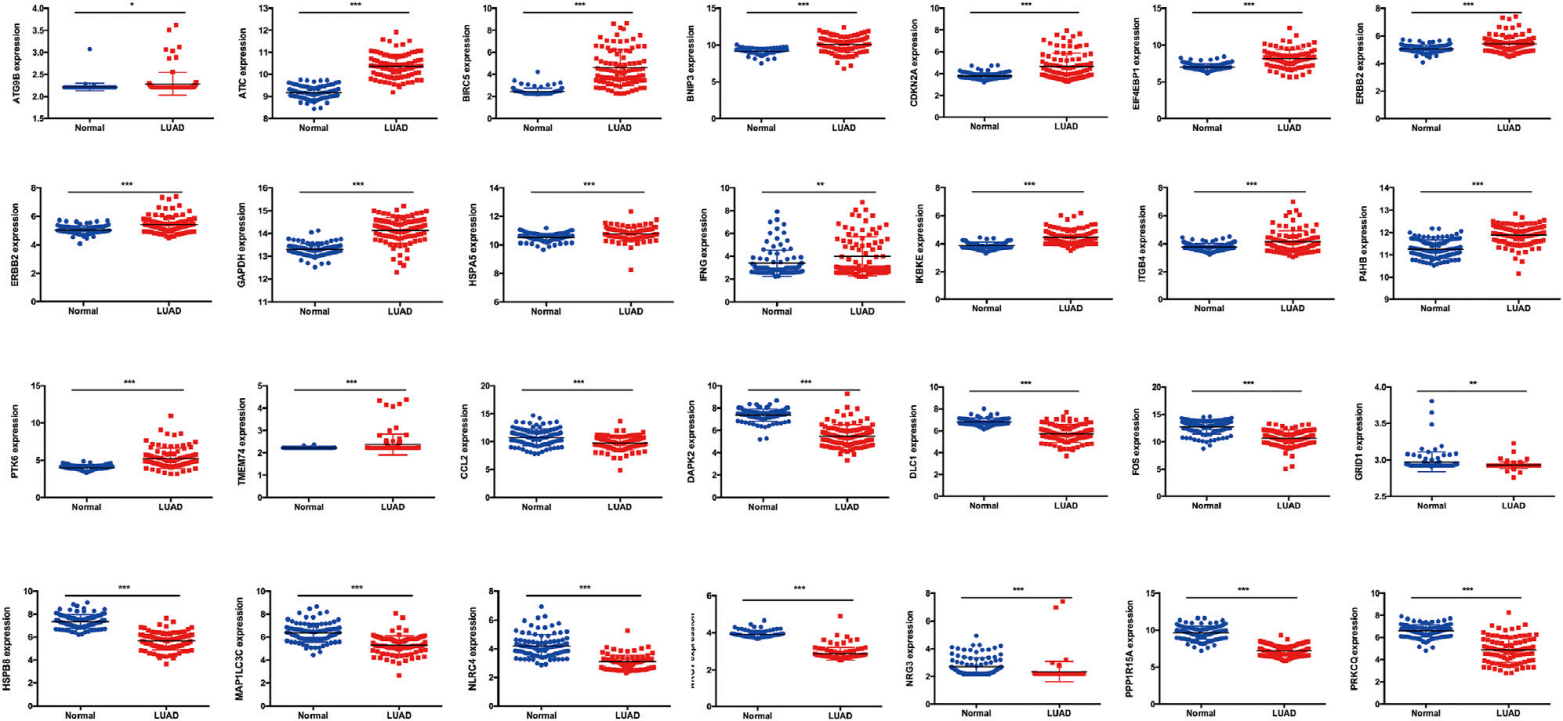
The validation of 28 DEARGs in LUAD using an independent cohort GSE72094, in which expression of 16 of 28 ARGs was significantly elevated and 12 genes was downregulated in LUAD tissues compared with adjacent normal lung tissues in GSE72094 (all *p* < 0.050). DEARGs, differentially expressed autophagy-related genes; LUAD, lung adenocarcinoma.

**FIGURE 7:**
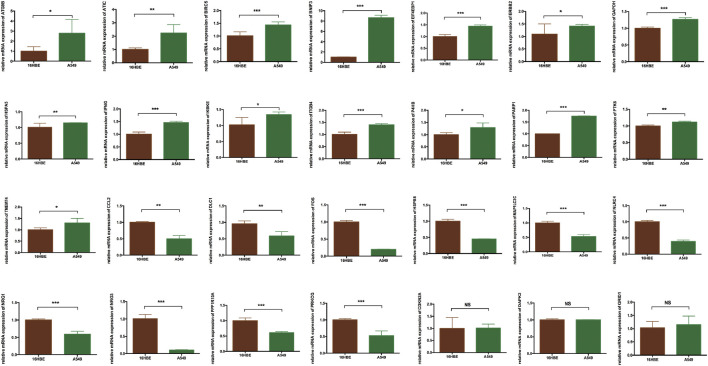
The validation of 28 DEARGs in LUAD using qRT-PCR, in which 25 of the 28 genes showed the significance in 16HBE compared with A549 cell lines (all *p* < 0.050), while three genes showed that there is no significance between 16HBE and A549 cell lines (all *p* > 0.050). DEARGs, differentially expressed autophagy-related genes; LUAD, lung adenocarcinoma; qRT-PCR, quantitative reverse transcription–polymerase chain reaction.

**FIGURE 8:**
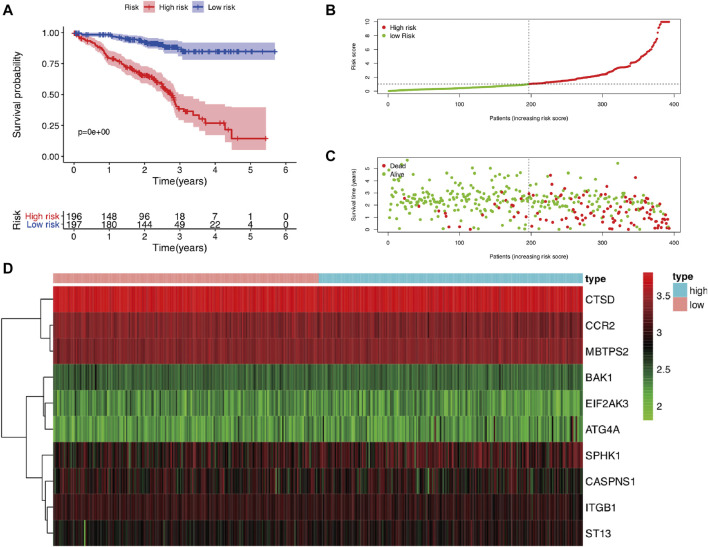
The validation of the prognostic risk model based on ARGs in LUAD using an independent cohort GSE72094. **(A)** Kaplan–Meier analysis of OS between high-risk and low-risk groups in LUAD patients based on risk scores according to 10 ARGs signatures. **(B)** Visualization of the risk score plot of high-risk and low-risk groups in LUAD patients. **(C)** Survival status was displayed in red and green dots based on the risk model as the risk score increasing in LUAD patients. **(D)** Two-dimensional hierarchical clustering of the significant 10 differentially expressed prognostic ARGs in high-risk and low-risk LUAD patients. ARGs, autophagy-related genes; lung adenocarcinoma; OS, overall survival.

To verify the risk model, the gene expression profile of GSE72094 was used for further analyses. The results showed that 28 DEARGs were pooled as differentially expressed in LUAD (Figure 6), in which the expression of 16 of 28 ARGs was significantly elevated and 12 genes were downregulated in LUAD tissues compared to adjacent normal lung tissues in GSE72094 (all *p* < 0.050), consistent with our previous results from TCGA database. For consensus clustering based on these 28 DEARGs, qRT-PCR was also performed. As we expected, the results showed that 25 of the 28 genes were consistent with the above results. Three genes were not significantly different between the 16HBE and A549 cell lines (Figure 7 **)**, which could be caused by the differences between tissues and cell lines. Furthermore, univariate Cox regression analysis showed that 40 genes were pooled as prognostic factors (Supplementary Table S1), and multivariate Cox regression analysis results suggested that 17 genes represented independent prognostic indicators, including 10 genes (ATG4A, BAK1, CAPNS1, CCR2, CTSD, EIF2AK3, ITGB1, MBTPS2, SPHK1, ST13) mentioned above (Supplementary Table S1). Then, 393 LUAD patients in GSE72094 were divided into low-risk (*n* = 196) and high-risk groups (*n* = 197) according to the previous formula. Survival analysis results also validated that the low-risk group exhibited significantly better prognosis than the high-risk group (*p* < 0.001) (Figure 8A). The risk score plot, survival time, and status plot are also shown in Figures 8B,C. Finally, the heat map plot was visualized to further illustrate the distribution of 10 prognostic ARGs between the high-risk and low-risk groups (Figure 8D).

### Survival Analysis of the Autophagy-Related Genes Expression Pattern and Clinicopathological Variables in Lung Adenocarcinoma

**FIGURE 9:**
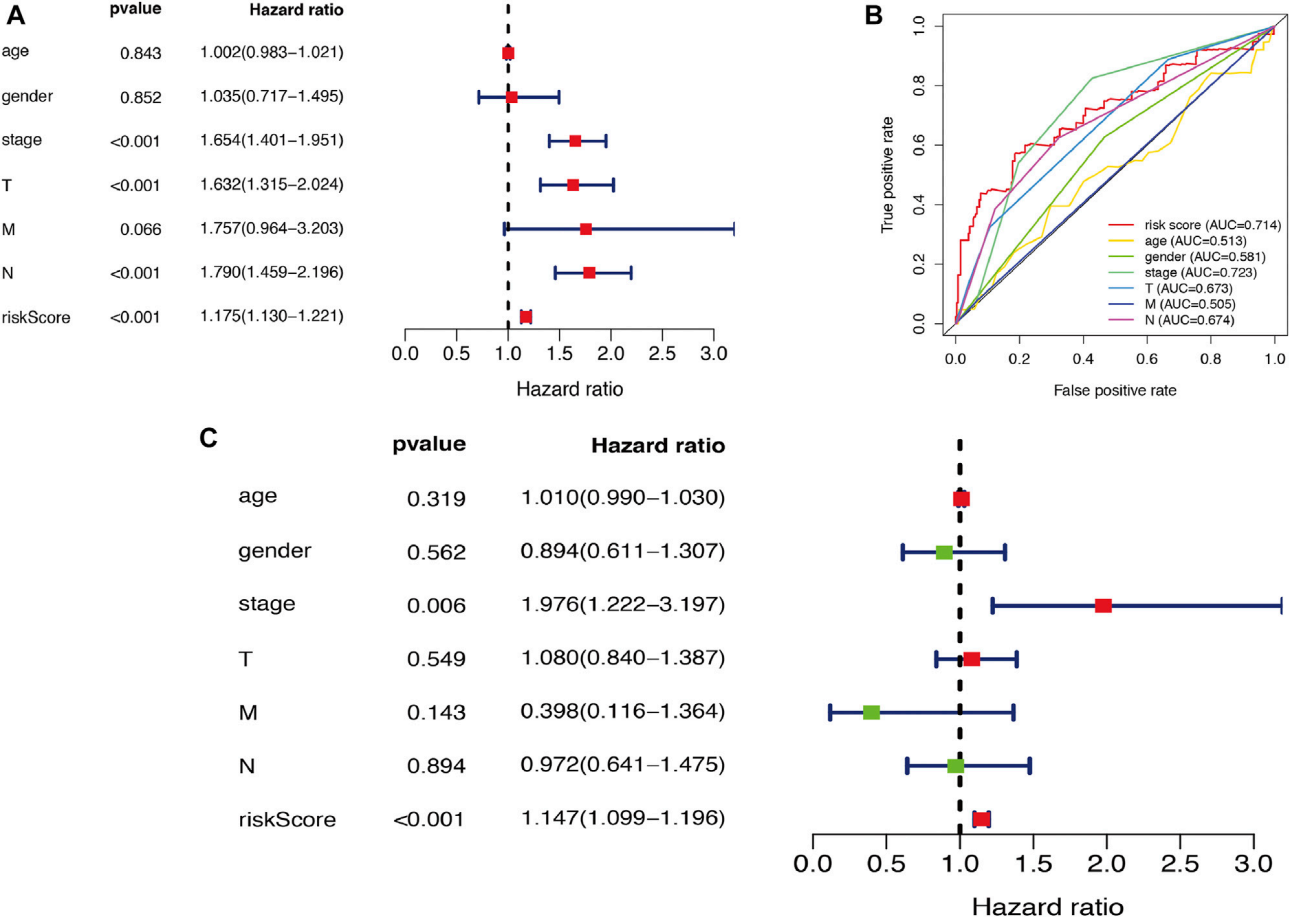
Cox regression analysis of risk model and clinicopathological variables in LUAD. **(A)** Visualization of the forest map with single factor independent prognostic analysis. **(B)** The ROC analysis of OS for the risk model signature and the clinicopathologic variables. **(C)** Visualization of the forest map with multifactor independent prognostic analysis. LUAD, lung adenocarcinoma; ROC, receiver operating characteristic; OS, overall survival.

In this study, univariate and multivariate Cox regression analyses were performed to explore the prognostic value of autophagy-related gene expression patterns and clinicopathological variables. Univariate Cox regression analysis results indicated that stage, pathological T stage, pathological N stage, and risk score were correlated with OS (all *p* < 0.001) (Figure 9A). Multivariate Cox independent prognostic analysis results indicated that stage and risk score represented independent prognostic factors in LUAD (*p* = 0.006 and *p* < 0.001, respectively) (Figure 9C). In addition, given that the ARGs had different values in this model, ROC curves of OS were used to determine the predictive performance of the 10 ARG risk patterns (Figure 9B). The AUC value of the risk score (marks the 10 ARG risk pattern) for OS was 0.714, which was significantly higher than that of age (AUC = 0.513), sex (AUC = 0.581), pathological T stage (AUC = 0.673), pathological N stage (AUC = 0.505), and pathological M stage (AUC = 0.674). These results indicated that the risk score had a better ability to predict survival in LUAD patients than other clinical factors.

### Correlation Analysis Between Autophagy-Related Genes and Clinicopathological Variables in Lung Adenocarcinoma

**FIGURE 10:**
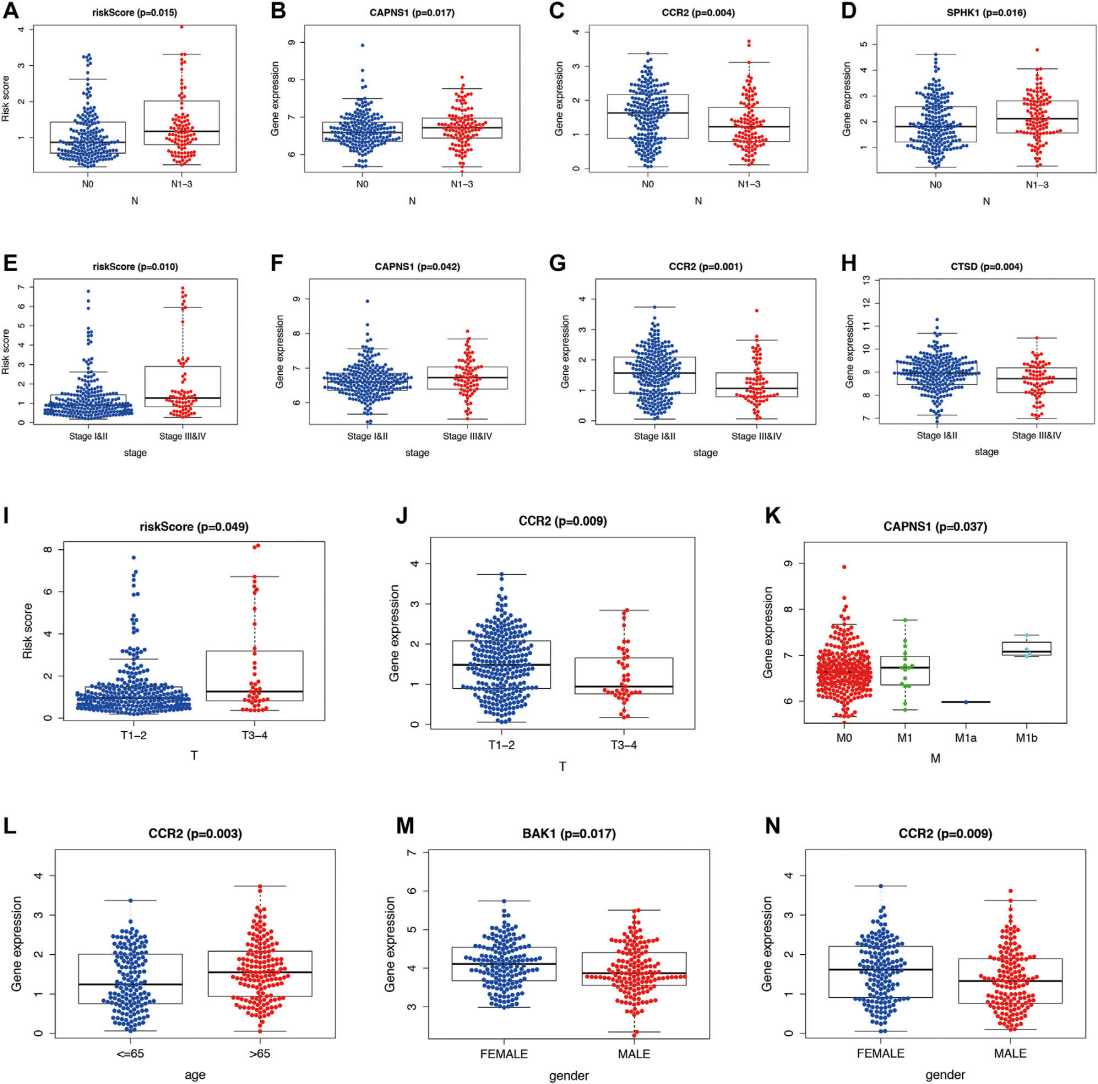
Comparison of ARGs and clinicopathological variables in LUAD. **(A–D)** Difference in the risk score and expression levels of ARGs between pathological N0 stage and pathological N1–N3 stages. **(E–H)** Difference in the risk score and expression levels of ARGs in stage I and II and stage III and IV. **(I,J)** Difference in the risk score and expression levels of ARGs in pathological T3–T4 stage and pathological T1–T2 stage. **(K)** Difference in the expression levels of ARGs in pathological M0 and M1 stage. **(L)** Difference in the expression levels of ARGs in aged ≤65 and >65 years. **(M,N)** Difference in the expression levels of ARGs in gender (male and female). ARGs, autophagy-related genes; LUAD, lung adenocarcinoma.

After pooling 10 independent risk genes, we further performed correlation analysis between ARGs and clinicopathological variables, such as age, sex, stage, pathological T stage, pathological N stage, and pathological M stage. The risk score shows potential prognostic value because of its significant difference in LUAD patients with pathological N0 stage compared to pathological N1-N3 stages (*p* = 0.015) (Figure 10A), and expression levels of CAPNS1 (*p* = 0.017), CCR2 (*p* = 0.004), and SPHK1 (*p* = 0.016) showed the same results (Figures 10B–D). The risk score also exhibited potential prognostic value because of its significant difference in stage I and II LUAD patients compared to stage III and IV LUAD patients (*p* = 0.010) (Figure 10E), and the same results were observed in the expression levels of CAPNS1 (*p* = 0.042), CCR2 (*p* = 0.001), and CTSD (*p* = 0.004) (Figures 10F–H). In addition, the risk score and expression level of CCR2 were significantly different in LUAD patients between pathological T3–T4 stage and pathological T1–T2 stage (*p* = 0.049 and *p* = 0.009) (Figures 10I,J). For the pathological M stage, only CAPNS1 displayed a difference between M0 and M1 LUAD patients (*p* = 0.037) (Figure 10K). Expression levels of CCR2 were significantly different in LUAD patients aged ≤65 and >65 years (*p* = 0.003) (Figure 10L). Furthermore, sex was not a factor to be ignored and was associated with expression levels of BAK1 (*p* = 0.017) and CCR2 (*p* = 0.009) (Figures 10M,N). According to the results, five ARGs exhibited significant differences among clinicopathological variables.

### Validation of Five Autophagy-Related Genes Expression Levels and Their Relationship With Overall Survival in Lung Adenocarcinoma

Five ARGs were screened as prognostic genes in TCGA database, and we validated their expression levels using the online GEPIA tool. The results showed that BAK1 and SPHK1 were highly expressed (Supplementary Figures S1A,B), while CAPNS1, CCR2, and CTSD were downregulated (Supplementary Figures S1C–E) in LUAD patients compared to controls. Unfortunately, these differences were not statistically significant. However, these five genes not only had prognostic value but also showed significant differences among clinicopathological variables according to our previous results, which attracted our attention. Furthermore, we performed Kaplan–Meier survival analysis using the Kaplan–Meier plotter online tool to validate the prognostic value of five ARGs, four of which, BAK1 (*p* = 2E-04), CAPNS1 (*p* < 0.001), CCR2 (*p* = 4.5E-12), and SPHK1 (*p* = 1.4E-06), exhibited prognostic value (Supplementary Figures S1F–I). However, in contrast to our previous findings, CTSD (also named CLN10) expression levels seemed to have no effect on OS in LUAD (*p* = 0.37) (Supplementary Figure S1J). SPHK1, as an attractive gene, was selected for further specific study after considering its expression levels and prognostic value.

### SPHK1 Expression Level and its Associations With Overall Survival and Tumor Infiltrating Lymphocytes

**FIGURE 11:**
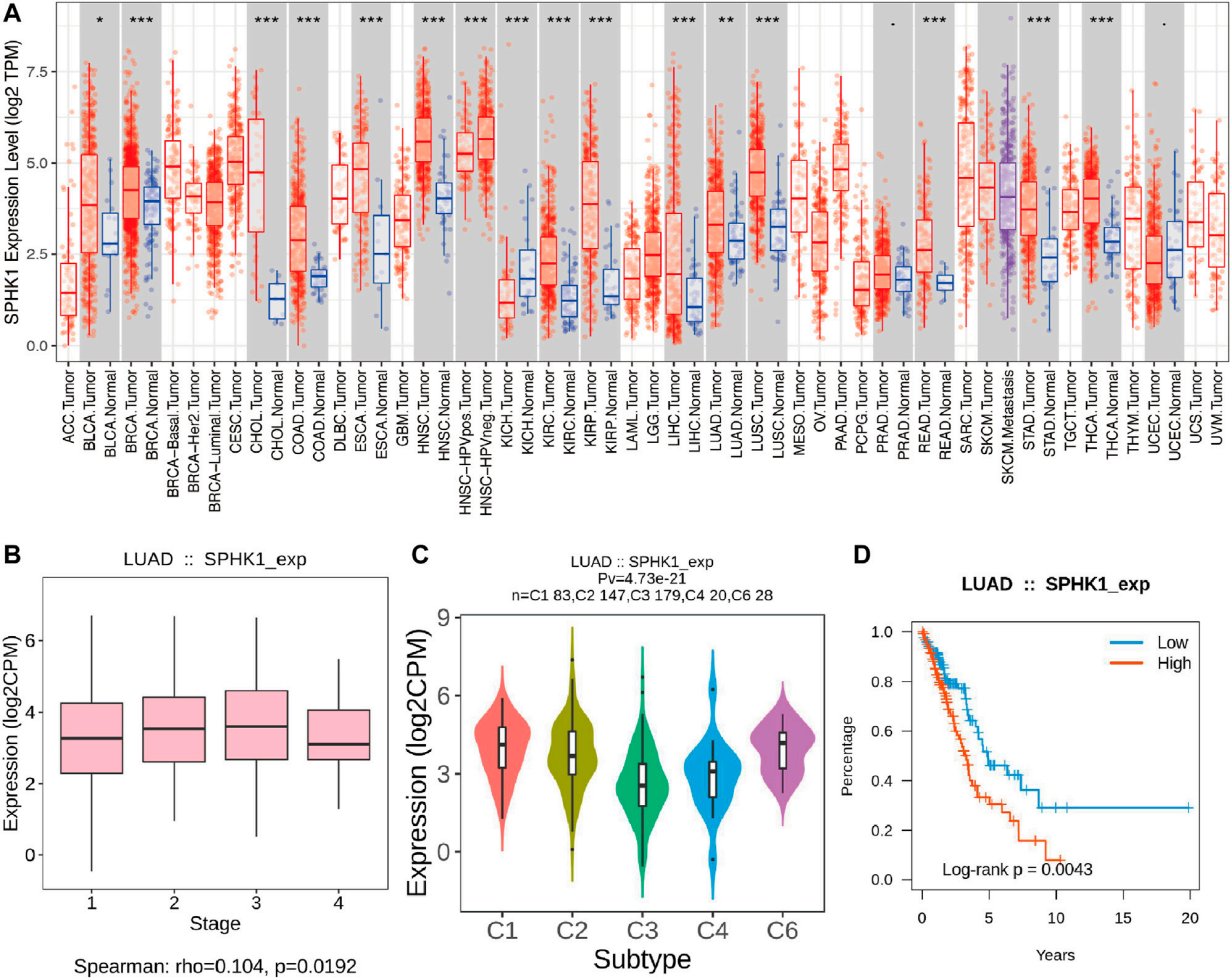
Validation of SPHK1 expression level and its associations with OS. **(A)** SPHK1 expression level in various cancers compared with normal tissues. **(B)** SPHK1 expression level in different stages of LUAD. **(C)** SPHK1 expression distribution across LUAD subtype. **(D)** Kaplan–Meier survival analysis by different SPHK1 expression level of LUAD. OS, overall survival; LUAD, lung adenocarcinoma.

TIMER and TISIDB were used to explore the relationship between SPHK1 and TILs. First, we validated the high expression level of SPHK1 (*p* < 0.010) (Figure 11A) and its expression level in different stages (*p* = 0.019) (Figure 11B). In addition, we further explored the SPHK1 expression distribution across LUAD subtypes (*p* = 4.73E-21) (Figure 11C). Consequently, we performed a Kaplan–Meier analysis between high and low SPHK1 expression levels, and the results showed that low expression levels of SPHK1 were associated with a better prognosis (*p* = 0.004) (Figure 11D). After validating the expression level of SPHK1 and its prognostic value, we also examined whether this phenomenon was related to TILs.

**FIGURE 12:**
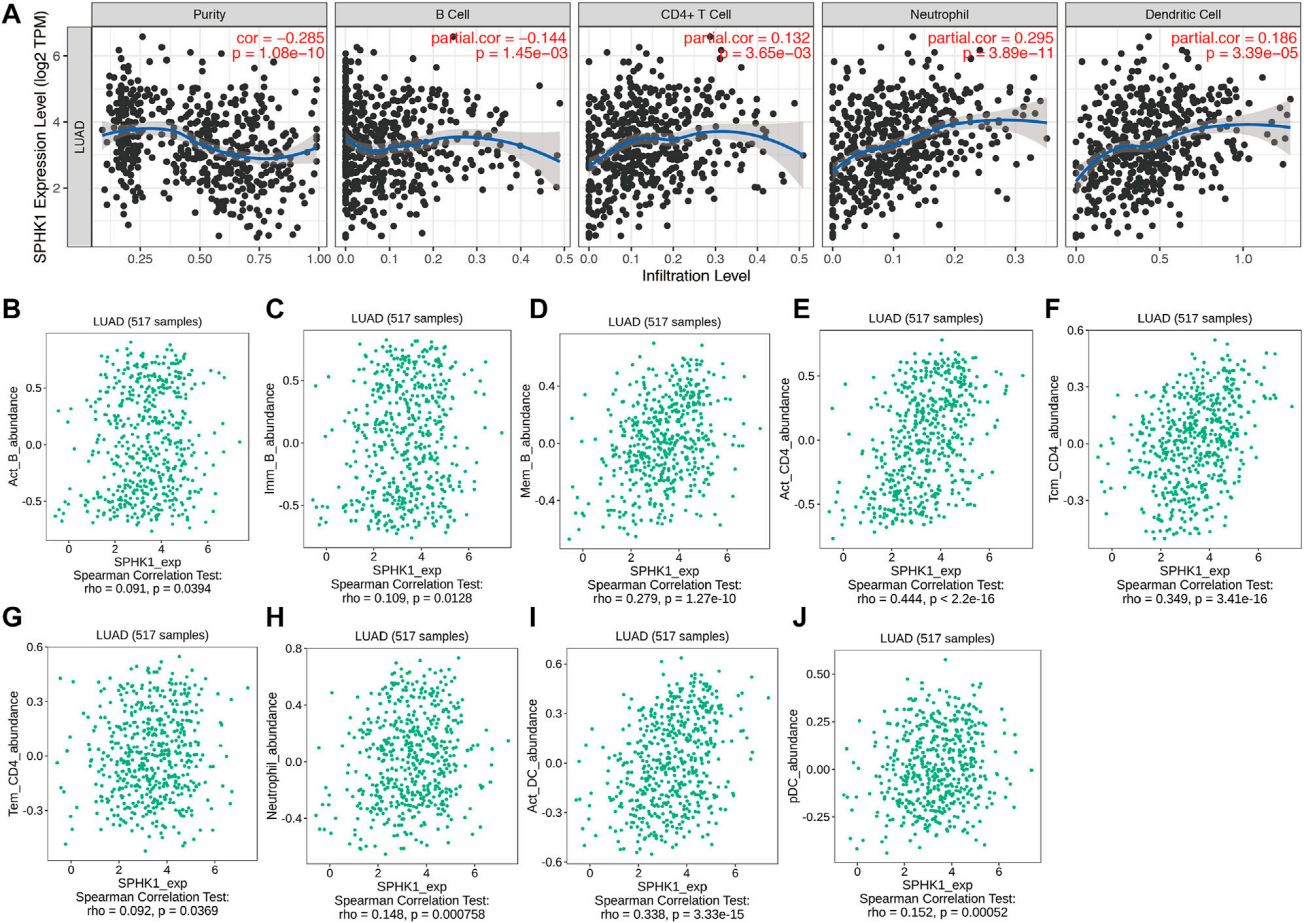
SPHK1 expression level associated with immune cell infiltration levels. **(A)** Correlation between SPHK1 expression levels and immune cell infiltration levels. Correlation between SPHK1 expression level and B Cell infiltration levels **(B–D)**, CD4^+^ T cells infiltration levels **(E–G)**, neutrophil cells infiltration levels **(H)**, dendritic cell infiltration levels **(I,J)**.

For further exploration, we conducted an integrated analysis to predict the potential biological roles of SPHK1 in TILs of LUAD. The results indicated that as tumor purity increased, the SPHK1 expression levels were negatively correlated with B cells (*r* = −0.144, *p* = 1.45E-03) and positively correlated with CD4^+^ T cell (*r* = 0.132, *p* = 3.65E-03), neutrophil (*r* = 0.295, *p* = 3.89E-11), and dendritic cell (*r* = 0.186, *p* = 3.39E-05) infiltration levels in the TIMER database (Figure 12A). Because data from one database seem to lack persuasiveness, we also validated the correlation between SPHK1 expression levels and immune cell infiltration using the TISIDB database. Consistent with our previous findings, except for the correlation between the SPHK1 expression level and B cell infiltration levels, SPHK1 expression levels were positively correlated with B cells (*r* = 0.091, *p* = 0.039; *r* = 0.109, *p* = 0.013; *r* = 0.279, *p* = 1.27E-10) (Figures 12B–D). The results showed that SPHK1 was positively correlated with CD4^+^ T cell (*r* = 0.444, *p* < 2.2E-16; *r* = 0.349, *p* = 3.41E-16; *r* = 0.092, *p* = 0.037) (Figures 12E–G), neutrophil (*r* = 0.148, *p* < 0.001) (Figure 12H), and dendritic cell (*r* = 0.338, *p* = 3.33E-15; *r* = 0.152, *p* = 0.001) infiltration levels, consistent with previous results.

### Associations Among Overall Survival, Somatic Copy Number Variation of SPHK1 and Tumor Infiltrating Lymphocytes Levels

**FIGURE 13:**
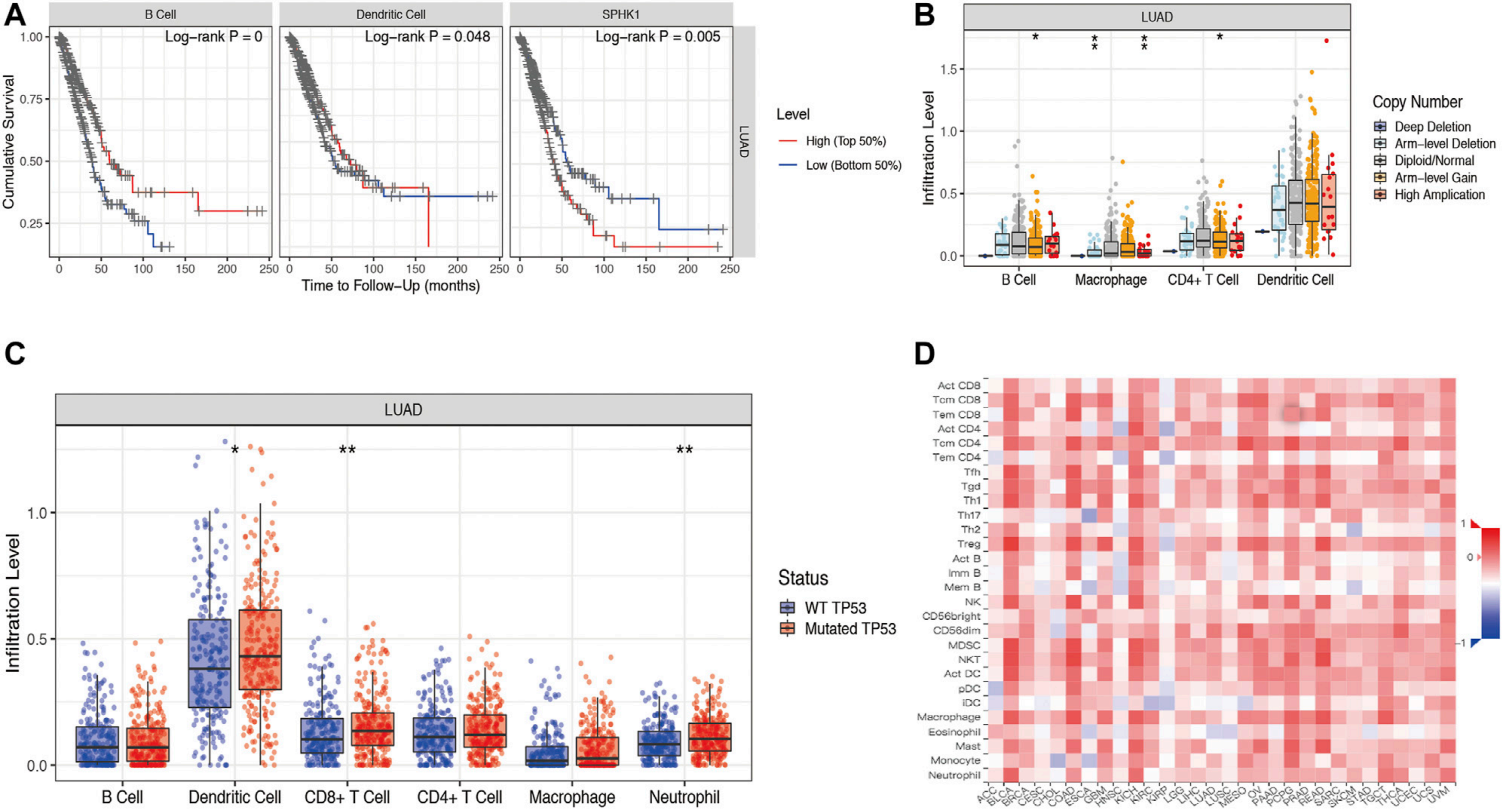
Associations among OS, SCNA of SPHK1, and TIL levels. **(A)** Cumulative survival rates between low and high immune cell (B cell and dendritic cell) infiltration; Kaplan–Meier survival analysis by different SPHK1 expression levels of LUAD. **(B)** Correlation between SCNA level of SPHK1 and immune cell infiltration level. **(C)** TP53 mutation rates were associated with CD8^+^ T cell, neutrophil, and dendritic cell infiltration levels. **(D)** Heat map analysis of SPHK1 expression level and tumor-infiltrating lymphocytes. OS, overall survival; SCNA, somatic copy number variation; TILs, tumor-infiltrating lymphocytes; LUAD, lung adenocarcinoma.

According to our previous results, we found that SPHK1 expression levels were correlated with B cell, CD4^+^ T cell, neutrophil, and dendritic cell infiltration levels, which may be the reason that differentially expressed SPHK1 has a different prognosis. Therefore, we first investigated the prognosis between high and low TIL expression levels. Kaplan–Meier analysis results revealed that cumulative survival rates between low and high B cell (*p* < 0.001) and dendritic cell (*p* = 0.048) infiltration levels were significantly different, as were the different expression levels of SPHK1 (*p* = 0.005) (Figure 13A). The SCNA module provides a comparison of the abundance of TILs among tumors with different somatic copy number aberrations for SPHK1. Therefore, we first explored the association between the SCNA level of SPHK1 and immune cell infiltration. We found that the B cell infiltration level was associated with arm-level gain (*p* < 0.050), macrophage infiltration level was associated with arm-level deletion and high amplification (both *p* < 0.010), and CD4^+^ T cell infiltration level was associated with arm-level gain (*p* < 0.050). For the dendritic cell infiltration level, there seemed to be no relationship with the SCNA level of SPHK1 (Figure 13B). Furthermore, TP53 mutation is one of the most common LUAD mutations and was correlated with CD8^+^ T cell (*p* < 0.010), dendritic cell (*p* < 0.050), and neutrophil (*p* < 0.010) infiltration levels (Figure 13C). Heat map analysis between SPHK1 expression levels and tumor-infiltrating lymphocytes in the TISIDB database was also performed and is displayed in Figure 13D, which is consistent with our previous results using the TIMER database.

## Discussion

In our current study, the limma package in R software was first used to identify DEARGs, and then functional enrichment analysis was performed to further refine the potential roles of DEARGs. Before performing Cox regression analysis, we classified LUAD cases into two subtypes according to consensus clustering based on the 28 prognostic ARGs. In addition, Kaplan–Meier analysis results showed differential prognoses between cluster 1 and cluster 2. Therefore, according to these two clusters, DEGs of each cluster were identified. Subsequently, univariate and multivariate Cox regression analyses were used to obtain the prognostic ARGs. After we overlapped the DEGs of clusters and the prognostic ARGs, 10 genes were selected for further study, and a risk model based on these genes was constructed to evaluate whether these genes could serve as independent prognostic factors for LUAD patients in TCGA database and performed subsequent validation using GSE72094. After we explored the associations between ARGs and clinicopathological variables, five ARGs were identified as prognostic genes. Then, we validated the expression levels of five ARGs and their relationships with OS in LUAD. SPHK1 was extracted for further study. In addition, the TIMER and TISIDB databases were used to further explore the correlation analysis between immune cell infiltration levels and the risk score as well as clinicopathological variables in the predictive risk model. In brief, a risk model associated with ARGs was constructed for monitoring immune cell infiltration levels and estimating the prognosis of LUAD.

Autophagy underlies the initiation, progression, and metastasis of various cancers, including LUAD, while aberrantly regulated autophagy affects the prognosis of LUAD, but the mechanisms are less well defined. Autophagy maintains cellular homeostasis by engulfing cytoplasmic proteins, complexes, or organelles within the autophagosome (Dikic et al., 2010; Macintosh and Ryan, 2013). Autophagosomes are cytoplasmic double-membraned vesicles that can be transported and fused with lysosomes to generate autolysosomes (Galluzzi et al., 2015). Autophagy has been reported to be associated with tumorigenesis (Martinet et al., 2009; Levy et al., 2017). Over the past few years, many studies have elucidated that autophagy participates in the development and progression of various diseases (Liu et al., 2017; Yao et al., 2018). In brief, autophagy exerts dual functions in tumorigenesis, including both positive and negative effects. Positive effects include autophagy clearing damaged proteins and organelles during the early stages of the tumor to inhibit tumor development (White et al., 2010). Negative effects are involved in the advanced stages of tumorigenesis, and autophagy promotes rapid growth of tumor cells by degrading and recycling damaged or aged organelle components (Janku et al., 2011). Furthermore, mechanisms related to autophagy have also been investigated in many studies (Chung et al., 2017; Bai et al., 2019; Chen et al., 2020; Liu et al., 2020; Peng et al., 2020). For example, PTBP1 promotes the growth of cancer cells through the PTEN/Akt pathway and autophagy in breast cancer (Wang et al., 2018). In addition, ATG5 and ATG7 regulate autophagy, apoptosis, and the cell cycle through PERK signaling, which is a vital UPR branch pathway (Zheng et al., 2019a). ER stress and autophagy are reportedly involved in the apoptosis of lung cancer (Shi et al., 2016). Interestingly, both ER stress and PERK signaling could be connected to autophagy in the presence of ATG5 and ATG7, revealing that the interplay among these different mechanisms should also be evaluated in further studies. Naturally, ARGs have also attracted increasing attention for their significance to the development of various cancers (Gu et al., 2016; Lin et al., 2018; Jiang et al., 2019; Lin et al., 2020). However, next-generation sequencing associated with ARGs and the establishment of a predictive risk model has not been well elucidated thus far. In addition, owing to the worse prognosis of LUAD, it is vital to identify novel prognostic biomarkers based on different methods.

In recent years, the development of potential prognostic biomarkers associated with ARGs to reveal prognosis has rapidly emerged. One study proposed an ARG prognostic signature and divided all patients into high-risk and low-risk groups, and the author concluded that the autophagy-related gene prognostic signature was a promising independent biomarker for monitoring the outcomes of serous ovarian cancer (An et al., 2018). Another eight ARGs (BCL2, BIRC5, EIF4EBP1, ERO1L, FOS, GAPDH, ITPR1, and VEGFA) were explored, and the author found that these genes not only were significantly associated with overall survival but also could predict distant metastasis-free survival in breast cancer (Gu et al., 2016). In the present study, we also identified 10 ARGs using Cox regression analysis and consensus clustering, and the coefficient values and gene expression values were further used to explore the risk score of each gene. According to the median risk score, all LUAD patients were divided into high-risk and low-risk groups. Survival analysis suggested that the low-risk group exhibited a significantly better prognosis than the high-risk group. Therefore, these 10 genes could serve as independent prognostic indicators and were selected for further exploration of the prognostic pattern in LUAD.

To investigate these 10 genes, correlation analysis was performed, and we found that only five (BAK1, CAPNS1, CCR2, CTSD, and SPHK1) of 10 genes were correlated with clinicopathological variables and prognosis in LUAD patients. BAK1 is associated with the development of oral squamous cell carcinoma and could serve as a prognostic biomarker in that malignancy (Baltaziak et al., 2004; Coutinho-Camillo et al., 2010). CAPNS1 has been explored as a crucial protein that could promote metastasis of hepatocellular carcinoma (Dai et al., 2014). Elevated expression levels of CCL2 were found to be correlated with tumor-associated macrophage accumulation, and both factors conveyed a poor prognosis in esophageal carcinogenesis (Yang et al., 2020). CTSD is one of the pivotal orchestrators in the occurrence and development of tumors, and its inhibition could increase autophagosome formation and decrease the formation of autolysosomes at the same time (Zheng et al., 2020). In this study, correlation analysis results suggested that these genes were correlated with age, sex, stage, pathological T stage, pathological N stage, or pathological M stage. Kaplan–Meier survival analysis suggested that CTSD (also called CLN10) expression levels seem to have no effects on OS in LUAD.

After screening the validated prognostic ARGs, SPHK1, as an attractive gene, was selected for further specific study after considering its expression level and prognostic value. Autophagy is involved in immune cell infiltration levels, and ARGs can affect immune responses. Autophagy is very important for the major functions of neutrophils, such as differentiation, phagocytosis, cytokine production, degranulation, and cell death (Germic et al., 2019). It has been demonstrated that enhanced autophagy or lysosome function in immune evasion could be achieved by selecting targets of MHC-I molecules for degradation, which could provide a therapeutic strategy against pancreatic ductal adenocarcinoma (Yamamoto et al., 2020). Various previous clinical studies have suggested that immune cell infiltration levels have a major impact on the clinical outcomes of several cancers (Bates et al., 2006; Galon et al., 2006; Johnson et al., 2000; Al-Shibli et al., 2008). SPHK1 is also known as SPHK. As characterized in a gene database (https://www.ncbi.nlm.nih.gov/gene/), this protein and its product S1P play a key role in immune processes. It has been observed that hypoxia-induced SPHK1 expression and its downstream S1P signaling promote ovarian cancer progression, and elevated expression levels of SPHK1 or S1P are sensitive to the cytotoxic effects of metformin (Hart et al., 2019). In addition, an increasing number of studies have shown the potential therapeutic value of SPHK1, which provides new strategies for cancer treatment to improve the prognosis of cancer patients (Plano et al., 2014; Wang et al., 2019c; Sukocheva et al., 2020). Another study suggested that enhanced SPHK activity promotes cell survival in Jurkat T cells in response to ceramide- or Fas-induced apoptosis (Zheng et al., 2019b). In this study, we found that SPHK1 expression is associated with the prognosis of LUAD. Furthermore, the SPHK1 expression levels were positively correlated with B cell, CD4^+^ T cell, neutrophil, and dendritic cell infiltration levels. In contrast, Kaplan–Meier analysis results showed that only B cell and dendritic cell infiltration levels were significantly correlated with prognosis.

In this study, gene expression profiles from TCGA database in LUAD were selected, and a risk model based on ARGs was established to predict the prognosis of LUAD. Subsequently, their prognostic value, association with clinicopathological variables, and the interesting association between SPHK1 and immune cell infiltration levels were validated. Compared to previous studies, the current study first constructed a risk model based on 10 ARGs, and we validated their prognostic value. Finally, five pooled ARG expression signatures were used as independent prognostic factors in patients with LUAD, which may provide new insight for monitoring and predicting the prognosis of LUAD patients.

## Data Availability

The datasets presented in this study can be found in online repositories. The names of the repository/repositories and accession number(s) can be found in the article/Supplementary Material.

## Supplementary Material

Supplementary Figure S1 Expression levels of five prognostic ARGs and prognosis in LUAD patients according. **(A,B)** BAK1 and SPHK1 were highly expressed in LUAD compared with normal lung tissues. **(C–E)** CAPNS1, CCR2 and CTSD were down regulated in LUAD compared with normal lung tissues. **(F–I)** BAK1, CAPNS1, CCR2 and SPHK1 showed their prognostic value. **(J)** CTSD (also named CLN10) expression level seems have no relationship with OS in LUAD. LUAD, lung adenocarcinoma; OS, overall survival.

## Abbreviations

ARGs, autophagy-related genes; DEARGs, differentially expressed autophagy-related genes; GEPIA, Gene Expression Profiling Interactive Analysis; GO, Gene ontology; HADb, Human Autophagy Database; HRs, hazard ratios; KEGG, Kyoto Encyclopedia of Genes and Genomes; LUAD, lung adenocarcinoma; LUSC, lung squamous cell carcinoma; OS, overall survival; ROC, Receiver Operating Characteristic; SCNA, somatic copy number variation; TCGA, the Cancer Genome Atlas; TILs, tumor infiltrating lymphocytes.
