# Supplementary figures and images for: Identification of a Five Autophagy Subtype-Related Gene Expression Pattern for Improving the Prognosis of Lung Adenocarcinoma
Source: Front Cell Dev Biol. 2021 Nov 18;9:756911. doi: 10.3389/fcell.2021.756911 (PMC8636677; doi:10.3389/fcell.2021.756911)

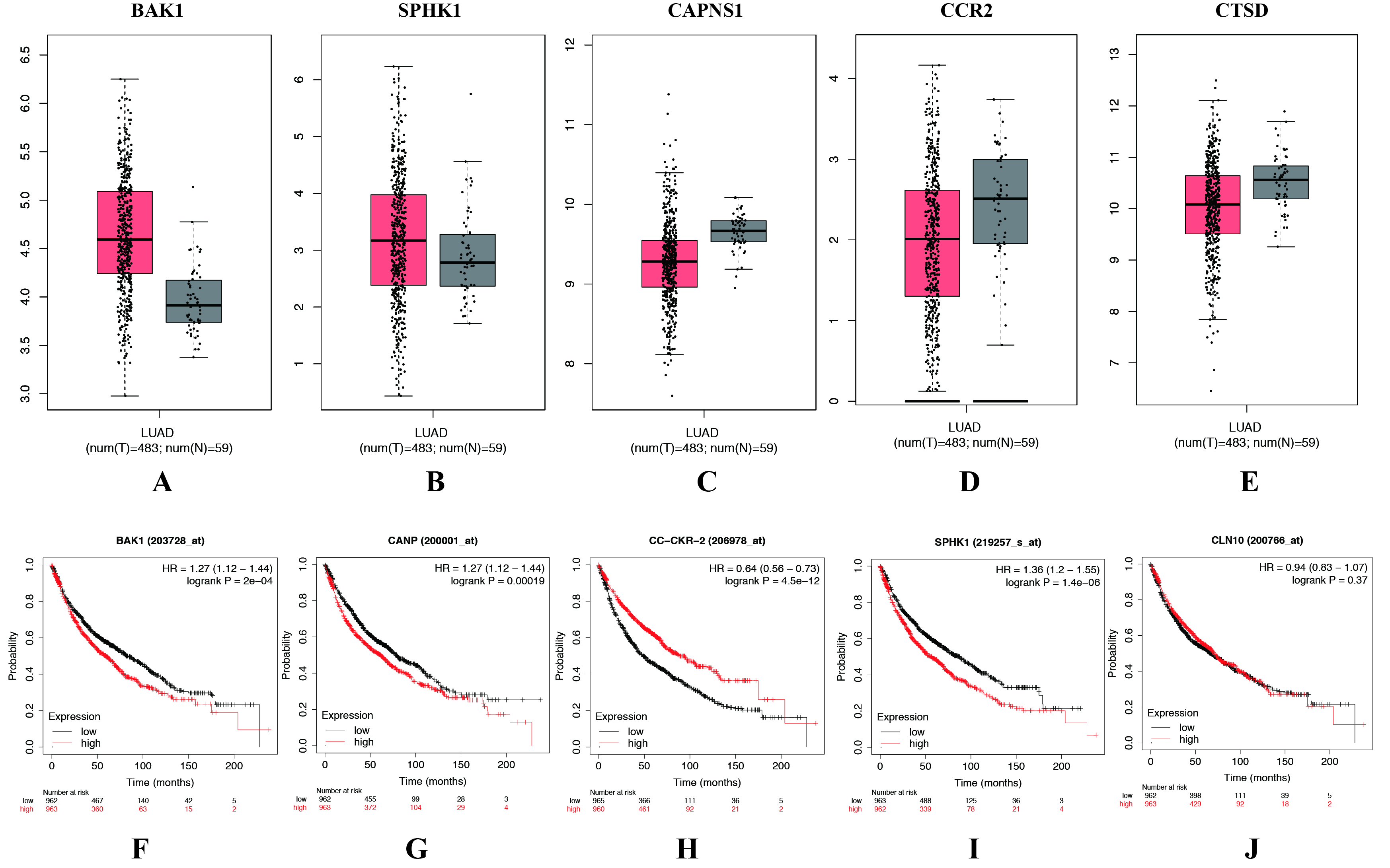

Supplement: Supplementary file 1 [file Image1.TIF]
